# Unscented Particle Filtering for Estimation of Shipboard Deformation Based on Inertial Measurement Units

**DOI:** 10.3390/s131115656

**Published:** 2013-11-15

**Authors:** Bo Wang, Xuan Xiao, Yuanqing Xia, Mengyin Fu

**Affiliations:** School of Automation and National Key Laboratory of Intelligent Control and Decision of Complex Systems, Beijing Institute of Technology, Beijing 100081, China; E-Mails: wb1020@bit.edu.cn (B.W.); xia_yuanqing@bit.edu.cn (Y.X.); fumy@bit.edu.cn (M.F.)

**Keywords:** shipboard deformation, inertial measurement unit, unscented particle filter, attitude plus angular rate match mode

## Abstract

Shipboard is not an absolute rigid body. Many factors could cause deformations which lead to large errors of mounted devices, especially for the navigation systems. Such errors should be estimated and compensated effectively, or they will severely reduce the navigation accuracy of the ship. In order to estimate the deformation, an unscented particle filter method for estimation of shipboard deformation based on an inertial measurement unit is presented. In this method, a nonlinear shipboard deformation model is built. Simulations demonstrated the accuracy reduction due to deformation. Then an attitude plus angular rate match mode is proposed as a frame to estimate the shipboard deformation using inertial measurement units. In this frame, for the nonlinearity of the system model, an unscented particle filter method is proposed to estimate and compensate the deformation angles. Simulations show that the proposed method gives accurate and rapid deformation estimations, which can increase navigation accuracy after compensation of deformation.

## 1. Introduction

Inertial measurement units (IMUs) are widely used in ships, aircraft and land vehicles to provide navigation information such as attitude, velocity and position [1,2]. The inertial navigation system (INS), which is based on an IMU, is more reliable for its autonomy and independence [3], compared to other navigation system, such as GPS [4]. In some vehicles, like ships, more than one INS is mounted to supply information to different onboard facilities and devices, such as the observing, sighting and lunching systems. In this circumstance, the INS which is mounted on the center of the vessel providing navigation information to the whole ship is called the main INS, while others are called slave INSs. During operation, the main INS will provide high accuracy initial navigation information, such as heading and attitude [5], to the slave INSs.

Shipboard is not an absolute rigid body. Many factors could cause deformations which lead to larger misalignment angles between the coordinates of the main INS and slave INSs [6,7]. Generally, these coordinates could be matched with a certain accuracy during ship construction, however, misalignment errors could occur because of environmental influences, such as waves, temperature, loads and shocks when the ship is launched in the sea. There are two kinds of deformation, one is the long term static deformation caused by structure aging, and the other is a short term dynamic deformation. Although the latter has limited effect on the general application of equipment, for high-accuracy INS, such an effect could cause large navigation errors, especially for the aided alignment between the main and slave INSs [8–10].

**Figure 1.**
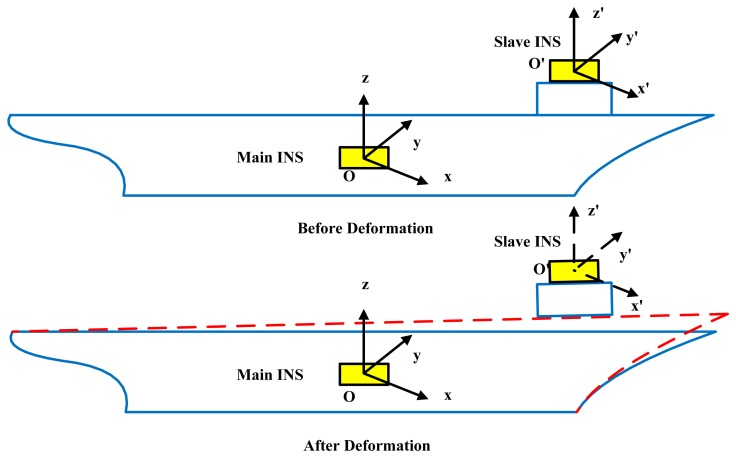
Dynamic deformation of a ship.

When deformation occurs, the attitude information provided by the main INS contains not only the attitude angle between navigation frame and the local coordinate frames of every equipment, but also the deformation angle between the installation spot of the main and slave INS [11]. This error will cause serious reduction of measurement and navigation accuracy. No matter how accurate the data the main INS provides, the navigation accuracy of the slave INS will be diluted by the deformation error. Therefore, for equipment distributed on the deck, the dynamic deformation will cause large errors in the aided alignment of INSs. It is very difficult to build an accurate analytical model of deformation. Because of structure, load distribution and operation conditions, we can only use approximate models for deformation estimation to enhance navigation accuracy [12]. In order to increase the observability, both the deterministic observability and the random observability should be considered. The random observability is focused on the effect of observation errors on the system state observability [13]. For aided alignment, the main source of observation errors is the relative movement between the main INS and slave INSs, which is induced by dynamic deformation (as shown in Figure 1). Because of this complex influence, the large angle deformation has strong nonlinearity, randomness and uncertainty. Besides, the influence of the complex sea surface environment is non-Gaussian. Therefore, an effective filter method for nonlinear and non-Gaussian systems is very necessary in shipboard deformation estimation [14–16].

For nonlinear Gaussian systems, the Extended Kalman Filter (EKF) is a widely used method [17,18]. However, it will induce large estimation errors for nonlinear and non-Gaussian system estimation. The unscented Kalman Filter (UKF) uses a nonlinear model and an unscented transformation obtaining a set of sigma sample points to calculate mean value and variance of states with better estimation performance. UKF also assumes that the real distribution is Gaussian. When this assumption is not satisfied, the Particle Filter (PF) would be a better solution. PF utilizes Monte Carlo simulation to approximate the whole conditional probability distribution, which is suited for arbitrarily nonlinear and non-Gaussian distributions [19]. The basic idea is applying a series of weighted random sample points to represent the *a posteriori* probability density function, and obtain an estimation using these sample points and weights. If the sample points are sufficient, their statistical property approximates that of an *a posteriori* probability density. Therefore, such estimations only have theoretical significance. The standard particle filter uses a prior probability density as an important sampling function without considering the latest measurement information. Even when the noise in the measurement information is small, on the contrary, filter accuracy decreases or diverges with a heavy calculation burden. However, the UKF focuses on the transformation and improvement of the linear Kalman filter for nonlinear systems. The system should meet Gaussian distribution conditions, otherwise, the simple representation of the state probability distribution by mean and variance will cause poor performance [20].

In recent years, many improved PF methods were proposed, especially for important density function and resampling methods [21–23]. The Unscented Particle Filter (UPF) is a state estimation method for non-Gaussian nonlinear systems. It is a kind of particle filter using UKF to generate new particles and obtain a more important density function. The UPF method, which combines UKF and PF together, utilizes UKF as the sampling method for PF, in order to increase the accuracy and speed of the standard PF. In the meantime, it overcomes the limitation of the Gaussian distribution assumption of UKF states. The unscented transformation will give a deterministic *a posteriori* mean and variance of system states, while PF makes less of a limitation for the probability density of system states.

In this work, we consider the shipboard deformation as a nonlinear Markov process and build an attitude plus angular rate match mode of aided alignment considering deformation angles. The deformation angles are estimated by an UPF-based method. Then, simulations of the proposed estimation method and aided alignment method are given. The main contribution of this paper is proposing an aided alignment method with deformation estimated by an UPF method.

The rest of the paper is organized as follows: the nonlinear shipboard deformation model is presented in Section 2. In Section 3, an attitude plus angular rate match mode is proposed as an estimation frame. In Section 4, we propose a UPF-based method to estimate the deformation angles. In Section 5, simulations are used to verify the deformation estimation and compensation method. The conclusions are given in Section 6.

## 2. Nonlinear Model of Shipboard Deformation

Suppose the main INS is installed on point *O* on the ship. There is a base with center *O*′. The coordinate transformation from *Oxy*z to *O*′*x*′*y*′z′ could be represented by three rotations of pitch, roll and yaw from *O*′ to *O*.

For INS, the propagation of attitude error can be presented by a *ψ* angle equation or a *φ* angle equation [6,7]. The attitude error described by the *ψ* angle equation is not real and cannot be measured, while the *φ* angle equation one reflects the real misalignment angle with the velocity and position errors. By comparing the attitude equations of the main and slave INSs, we can obtain the attitude error which contains the shipboard deformation.

### 2.1. Attitude Error Propagation Model

The attitude error vector of the main and slave INS is *ψ_m_*, and the computation coordinate of the slave INS is *s*′. The differential attitude equations of main and slave INSs are as follows:

(1) $${\dot{C}}_{m}^{n}=C_{m}^{n}[{\hat{\omega }}_{nb}^{m}\times ]$$

(2) $${\dot{C}}_{s^{\prime }}^{n}=C_{s^{\prime }}^{n}[{\hat{\omega }}_{ns}^{s}\times ]$$

where $C_{m}^{n}$ is the attitude matrix of vehicle, $C_{s^{\prime }}^{n}$ is the coordinate transfer matrix from computation of the slave INS to main INS. $\left[{\hat{\omega }}_{nb}^{m}\times \right]$ is the skew symmetric matrix of ${\hat{\omega }}_{nb}^{m}$ [5].

For *ψ_m_*, we have:

(3) $$[\psi_{m}\times ]=I-C_{m}^{s^{\prime }}=I-C_{n}^{s^{\prime }}C_{m}^{n}$$

where *I* is a unit matrix. By solving differential Equation of (3), we have:

(4) $$\begin{array}{cc} \left[{\dot{\psi }}_{m}\right] & =[{\hat{\omega }}_{ns}^{s}\times ]C_{n}^{s^{\prime }}C_{m}^{n}-C_{n}^{s^{\prime }}C_{m}^{n}[(C_{s}^{m}{\hat{\omega }}_{nb}^{s})\times ]\\ & =[{\hat{\omega }}_{ns}^{s}\times ](I-[\psi_{m}\times ])-(I-[\psi_{m}\times ])[(I+[(\phi +\psi )\times ]){\hat{\omega }}_{nb}^{s})\times ]\\ & =[{\hat{\omega }}_{ns}^{s}\times ]-[{\hat{\omega }}_{nb}^{s}\times ]-[{\hat{\omega }}_{ns}^{s}\times ][\psi_{m}\times ]+[\psi_{m}\times ][{\hat{\omega }}_{ns}^{s}\times ]-[([(\phi +\psi )\times ]{\hat{\omega }}_{nb}^{s})\times ] \end{array}$$

where *φ* and *ψ* are the static and dynamic deformation, respectively.

In aided alignment, the output of the main INS is treated as a standard for the slave INSs. We ignore the positioning error and constant drift of gyro of the main INS and obtain:

(5) $$\left[{\hat{\omega }}_{ns}^{s}\times ]-[{\hat{\omega }}_{nb}^{s}\times ]=[\dot{\psi }\times ]+[\xi_{s}^{s}\times \right]$$

where $\xi_{s}^{s}$ is the gyro drift of the slave INS. Therefore, Equation (4) can be simplified as:

(6) $${\dot{\psi }}_{m}=-{\hat{\omega }}_{ns}^{s}\times (\psi_{m}-\psi -\phi )+\dot{\psi }+\xi_{s}^{s}$$

### 2.2. Velocity Error Propagation Model

The navigation equation of the slave INS is:

(7) $${\dot{V}}_{s}^{n}=C_{s^{\prime }}^{n}{\hat{f}}_{s}^{s}-({\hat{\omega }}_{en}^{n}+2{\hat{\omega }}_{ie}^{n})\times V_{s}^{n}+g_{s}^{n}-{\hat{\omega }}_{ie}^{n}\times ({\hat{\omega }}_{ie}^{n}\times R_{s}^{n})$$

where $V_{s}^{n}$is the computation velocity of the slave INS; ${\hat{f}}_{s}^{s}$is the specific force output of the slave INS accelerator; ${\hat{\omega }}_{en}^{n}$and ${\hat{\omega }}_{ie}^{n}$ are the angular velocity from the computational local level coordinates of the slave INS to Earth coordinates and from Earth coordinates to inertial coordinates, respectively; $g_{s}^{n}$ is the computational gravity of the slave INS; $R_{s}^{n}$ is the position vector from the slave INS to the Earth center.

As described before, the computational error of main INS can be ignored. Therefore, the navigation equation is:

(8) $${\dot{V}}_{m}^{n}=C_{m}^{n}f_{b}^{m}-(\omega_{en}^{n}+2\omega_{ie}^{n})\times V_{m}^{n}+g_{m}^{n}-\omega_{ie}^{n}\times (\omega_{ie}^{n}\times R_{m}^{n})$$

where $V_{m}^{n}$is the velocity of the vessel, $f_{b}^{m}$ is the projection of specific force to the slave INS coordinates, $\omega_{en}^{n}$is the angular velocity from the local level to the Earth coordinates, $\omega_{ie}^{n}$is the angular velocity from Earth to inertial coordinates, $g_{m}^{n}$is the projection of gravity to the local level coordinates, $R_{m}^{n}$ is the position vector from the main INS to the Earth center.

The specific force can be presented as:

(9) $${\hat{f}}_{s}^{s}=C_{m}^{s}f_{b}^{m}-\alpha_{r}^{s}-\alpha_{fs}^{s}+A_{s}$$

where $\alpha_{r}^{s}$is the lever arm acceleration, $\alpha_{fs}^{s}$is the deformation acceleration, and *A_s_* is the gyro error. We get:

(10) $$-(\alpha_{r}+\alpha_{fs})=\frac{d_{I}^{2}r}{dt}$$

where $r=R_{s}^{n}-R_{m}^{n}$, minus means inertial force. Expanding the last equation, we get:

(11) $$\frac{d_{i}r}{dt}=\frac{d_{e}r}{dt}+\omega_{ie}\times r=V_{r}+\omega_{ie}\times r$$

(12) $$\frac{d_{i}^{2}r}{dt}={\dot{V}}_{r}+(\omega_{in}+\omega_{ie})\times V_{r}+\omega_{ie}\times (\omega_{ie}\times r)$$

We can obtain the following equation:

(13) $$\begin{array}{ll} {\dot{V}}_{s}^{n}-{\dot{V}}_{m}^{n} & =C_{s^{\prime }}^{n}{\hat{f}}_{s}^{s}-C_{m}^{n}f_{b}^{m}-({\hat{\omega }}_{en}^{n}+2{\hat{\omega }}_{ei}^{n})\times V_{s}^{n}+(\omega_{en}^{n}+2\omega_{ie}^{n})\times V_{m}^{n}+g_{s}^{n}-g_{m}^{n}\\ & -{\hat{\omega }}_{ie}^{n}\times ({\hat{\omega }}_{ie}^{n}\times R_{s}^{n})+\omega_{ie}^{n}\times (\omega_{ie}^{n}\times R_{m}^{n}) \end{array}$$

The error of the computed Earth rotation angular rate caused by the position error in the system noise is considered. Because the position error is very small, the gravity can be regarded as the same. Therefore, we simplify Equation (13) as follows:

(14) $${\dot{V}}_{s}^{n}-{\dot{V}}_{m}^{n}=C_{s^{\prime }}^{n}{\hat{f}}_{s}^{s}-C_{m}^{n}f_{b}^{m}-({\hat{\omega }}_{en}^{n}+2\omega_{ie}^{n})\times V_{s}^{n}+(\omega_{en}^{n}+2\omega_{ie}^{n})\times V_{m}^{n}-\omega_{ie}^{n}\times (\omega_{ie}^{n}\times r^{n})$$

And then, we get the following equations:

(15) $$\begin{array}{ll} {\dot{V}}_{s}^{n}-{\dot{V}}_{m}^{n}-{\dot{V}}_{r}^{n} & =C_{m}^{n}(\psi_{m}-\psi -\phi )\times f_{b}^{m}-C_{s^{\prime }}^{n}(\alpha_{r}^{s}+\alpha_{fs}^{s}-A_{s})-(\delta \omega_{en}^{n}+\omega_{en}^{n}+2\omega_{ie}^{n})\times V_{s}^{n}\\ & +(\omega_{en}^{n}+2\omega_{ie}^{n})\times V_{m}^{n}-\omega_{ie}^{n}\times (\omega_{ie}^{n}\times r^{n})+C_{s}^{n}(\alpha_{r}^{s}+\alpha_{fs}^{s})+(\omega_{en}^{n}+2\omega_{ie}^{n})\times V_{r}^{n}\\ & +\omega_{ie}^{n}\times (\omega_{ie}^{n}\times r^{n}) \end{array}$$

Defining $\delta V=V_{s}^{n}-V_{m}^{n}-V_{r}^{n}$, and ignoring the second order small quantity, Equation (15) can be simplified as:

(16) $$\delta \dot{V}=C_{m}^{n}(\psi_{m}-\psi -\phi )\times (f_{b}^{s}-\alpha_{r}^{s}-\alpha_{fs}^{s})+\delta \omega_{en}^{n}\times V_{s}^{n}-({\hat{\omega }}_{en}^{n}+2{\hat{\omega }}_{ie}^{n})\times \delta V+C_{s^{\prime }}^{n}A_{s}$$

where *ψ_m_*, *δV* and *δω* can be measured as:

(17) $$\psi_{m}=\psi_{S}-\psi_{M}$$

(18) $$\delta \omega ={\hat{\omega }}_{ib}^{s}-({\hat{\omega }}_{ib}^{m}-\xi_{m})=\xi_{s}+\dot{\psi }+{\hat{\omega }}_{ib}^{m}\times (\phi +\psi )$$

(19) $$\delta V=V_{s}^{n}-V_{m}^{n}-V_{r}^{n}$$

$V_{r}^{N}$can be computed as:

(20) $${\hat{V}}_{r}=C_{s^{\prime }}^{n}{\hat{\omega }}_{is}^{s}\times r^{s}-{\hat{\omega }}_{ie}^{n}\times (C_{s^{\prime }}^{n}r^{s})$$

In practice, it can be rewritten as:

(21) $${\hat{V}}_{r}=C_{m}^{n}\omega_{ib}^{m}\times r^{m}-\omega_{ie}^{n}\times (C_{m}^{n}r^{m})$$

The differential equation of the static deformation angle is:

(22) $$\dot{\phi }=0$$

Assuming the dynamic shipboard deformation angle is:

(23) $$\psi ={\left[\begin{array}{ccc} \psi_{x} & \psi_{y} & \psi_{z} \end{array}\right]}^{T}$$

Dynamic deformation occurs when the ship is rotated by waves and winds. During navigation computation, we can use a second order Markov process driven by white noise to describe such a motion and assume the deformation processes of the three axes of the ship are independent. Let $\mu_{\theta }={\left[\begin{array}{ccc} \mu_{\theta_{x}} & \mu_{\theta_{y}} & \mu_{\theta_{z}} \end{array}\right]}^{T}$be the deformation angular rate of the body frame between the slave INS and the main INS caused by dynamic deformation, we have:

(24) $${\dot{\psi }}_{x}=\mu_{\theta_{x}};{\dot{\psi }}_{y}=\mu_{\theta_{y}};{\dot{\psi }}_{z}=\mu_{\theta_{z}}$$

(25) $$\begin{array}{c} {\dot{\mu }}_{\theta_{x}}=-\beta_{x}^{2}\psi_{x}-2\beta_{x}\mu_{\theta_{x}}+w_{\theta_{x}}\\ {\dot{\mu }}_{\theta_{y}}=-\beta_{y}^{2}\psi_{y}-2\beta_{y}\mu_{\theta_{y}}+w_{\theta_{y}}\\ {\dot{\mu }}_{\theta_{z}}=-\beta_{z}^{2}\psi_{z}-2\beta_{z}\mu_{\theta_{z}}+w_{\theta_{z}} \end{array}$$

where the variance of deformation angle is $\sigma^{2}={\left[\begin{array}{ccc} \sigma_{x}^{2} & \sigma_{y}^{2} & \sigma_{z}^{2} \end{array}\right]}^{T}$, $w={\left[\begin{array}{ccc} w_{\theta_{x}} & w_{\theta_{y}} & w_{\theta_{z}} \end{array}\right]}^{T}$is white noise with frequency spectrum density $Q_{w}={\left[\begin{array}{ccc} Q_{w_{x}} & Q_{w_{y}} & Q_{w_{z}} \end{array}\right]}^{T}$and *w*∼*N*(0, *Q_η_*). *β_i_*=2.146/ *τ_i_*, *τ_i_* is the correlation time of the deformation angles of the three axes, which is set according to the size of the ship. The relationship between *Q_w_*, σ and *β* is $Q_{w_{i}}=4\beta_{i}^{3}\sigma_{i}^{2}$, (*i*=*x*,*y*,*z*).

### 2.3. Numerical Simulation

**Figure 2.**
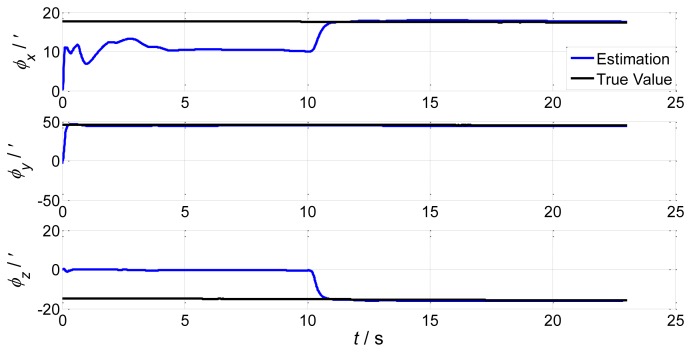
Attitude error estimations of the first set.

**Figure 3.**
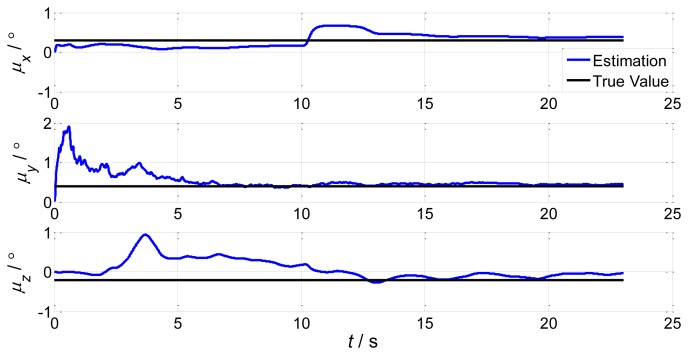
Installation error estimations of the first set.

**Figure 4.**
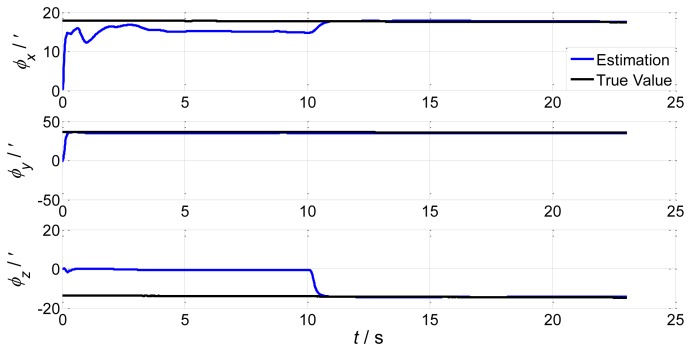
Attitude error estimations of the second set.

**Figure 5.**
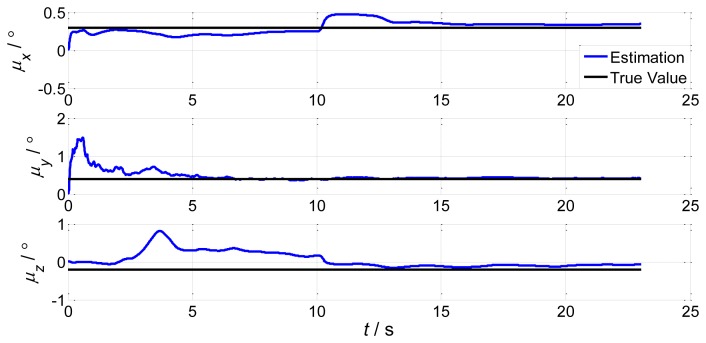
Installation error estimations of the second set.

During sailing, a lot of environmental disturbance will bring different levels of ship deformation and influence the aided alignment accuracy of the shipboard INS. In order to test the influence of deformation on aided alignment, we set the initial conditions of the INS as follows: the constant drift of the gyro is [0.01 0.01 0.01]*^T^* with °/*h* units; the white noise variance of the gyro is [0.003 0.003 0.003]*^T^* with °/*h* units; the constant bias of the accelerator is [100 100 100]*^T^* with μ*g* units; the white noise variance of the accelerator is [10 10 10]*^T^* with μ*g* units; the initial installation error angles are [0.3° 0.4° −0.2°]. The wave influence on the ship is small near the dock, and will get larger if the ship departs to sea. Therefore, in order to simulate a practical situation, we assume the ship is first in uniform linear motion for 10 s, then in sinusoidal motion because of the wave influence. According to the real ship and sea environment, simulation parameters are set as follows: the sinusoidal motion of the ship is along a rolling axis with amplitude 10° and period 5 s; the correlation time of the deformation process is 60 s; according to different sea conditions (severe and calm), we set two deformation amplitudes, the first is [1′ 31.2′ 5.2′] and the second is [0.69′ 17.4′ 3.1′]. Simulation results are as follows: the motion transformation of the ship leads to the spike at t = 10 s in Figures 2, 3, 4 and 5.

Simulation results demonstrate that the deformation angles have a certain influence on aided alignment, increasing with deformation. The installation error angles reflect the shipboard deformation estimated by the IMU. From comparison of Figures 3 and 4, we can conclude that once the sea conditions change to severe, the installation error angles obviously increase. Therefore, we must apply a proper match mode and estimation method of aided alignment to obtain an accurate estimation of shipboard deformation angles.

## 3. Attitude Plus Angular Rate Mode

This method compares the outputs of gyro and computed attitude angles between the main INS and slave INS to estimate the gyro drift and deformation. The system equation is as follows: first, we choose the state variable $X={\left[\begin{array}{lllll} \psi_{m}^{T} & \psi^{T} & {\dot{\psi }}^{T} & \phi^{T} & \xi_{s}^{sT} \end{array}\right]}^{T}$, then the state equation is:

(26) $$\dot{X}=F(t)X+G(t)w(t)$$

where:

(27) $$F(t)=\left[\begin{array}{lllll} A_{1} & A_{2} & A_{3} & A_{4} & A_{5}\\ 0 & 0 & A_{6} & 0 & 0\\ 0 & A_{7} & A_{8} & 0 & 0\\ 0 & 0 & 0 & 0 & 0\\ 0 & 0 & 0 & 0 & 0 \end{array}\right]$$

where, $A_{1}=-[{\hat{\omega }}_{ns}^{s}\times ]$, *A*_2_= −*A*_1_, *A*_3_, is a unit matrix, *A*_4_= −*A*_1_, *A*_5_, and *A*_6_ are unit matrices, $A_{7}=diag(-b_{x}^{2},-b_{y}^{2},-b_{z}^{2})$, *A*_8_=*diag*(−2μ*_x_*, −2μ*_y_*, −2μ*_z_*). *w*(*t*) is the unit white noise:

(28) $$G(t)={\left[\begin{array}{lllll} G_{1}(t) & 0 & 0 & 0 & 0\\ 0 & 0 & G_{2}(t) & 0 & 0 \end{array}\right]}^{T}$$

where *G*_1_(*t*) is the random drift of gyro, $G_{2}(t)=diag(2b_{x}\sqrt{D_{x}\mu_{x}},2b_{y}\sqrt{D_{y}\mu_{y}},2b_{z}\sqrt{D_{z}\mu_{z}})$

We choose the measurement $z(t)={\left[\begin{array}{cc} \psi_{m}^{T} & \delta \omega^{T} \end{array}\right]}^{T}$, and get the system measurement equation:

(29) z(t)=H(t)X+v(t)

## 4. UPF Method

In the PF method, we choose a set of discrete random sample points (particles) with weights to approximate the *a posteriori* probability density function and the estimation because in the standard PF, the importance sampling density function did not include the latest measurement information. There is large error between this sample and the one produced by the true *a posteriori* probability density function. The UPF method is an expanded PF with the same basic recurrence structure. Because UKF can provide accurate three order *a posteriori* variance and estimation of true state variance, this method obtains better importance particle density functions and is more suitable for the PF iteration frame. The re-sampling method can avoid sample impoverishment and increase the diversity, which resolves the degeneracy phenomenon as the key problem of PF. Based on the above idea, the UPF method can be described as follows:

Initialization: *t*=0
For *i*=1,2,…, *N*, *N* samples $x_{0}^{\left(i\right)}$ which follow the *a priori* distribution are generated:
  (30) $${\bar{x}}_{0}^{\left(i\right)}=E\left[x_{0}^{\left(i\right)}\right]$$
  (31) $$P_{0}^{\left(i\right)}=E\left[\left(x_{0}^{\left(i\right)}-{\bar{x}}_{0}^{\left(i\right)}\right){\left(x_{0}^{\left(i\right)}-{\bar{x}}_{0}^{\left(i\right)}\right)}^{T}\right]$$
  (32) $${\bar{x}}_{0}^{(i)a}=E\left[x_{0}^{(i)a}\right]={\left[\begin{array}{ccc} {\left({\bar{x}}_{0}^{\left(i\right)}\right)}^{T} & 0 & 0 \end{array}\right]}^{T}$$
  (33) $$P_{0}^{(i)a}=E\left[\left(x_{0}^{(i)a}-{\bar{x}}_{0}^{(i)a}\right){\left(x_{0}^{(i)a}-{\bar{x}}_{0}^{(i)a}\right)}^{T}\right]=\left[\begin{array}{ccc} P_{0}^{(i)a} & 0 & 0\\ 0 & Q & 0\\ 0 & 0 & R \end{array}\right]$$
For *t*=1,2,…

**Figure 6.**
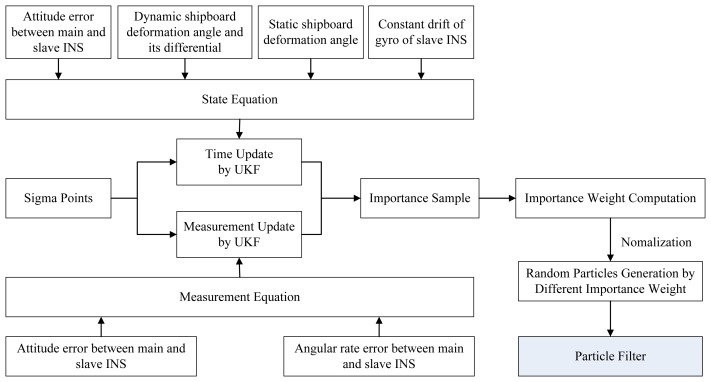
Flow chart of the proposed method.

(1) Importance sampling procedure:
  (i) For *i*=1,2,…, *N*:
    Using UKF to update new particles:
    Compute sigma points:
    (34) $$X_{t-1}^{(i)a}=\left[\begin{array}{cc} {\bar{x}}_{t-1}^{(i)a} & {\bar{x}}_{t-1}^{(i)a}\pm \sqrt{(n_{a}+\lambda )P_{t-1}^{(i)a}} \end{array}\right]$$
    Forward propagation particles (time update):
    (35) $$X_{t|t-1}^{(i)x}=f\left(X_{t-1}^{(i)x},X_{t-1}^{(i)v}\right)$$
    (36) $${\bar{x}}_{t|t-1}^{\left(i\right)}=\sum_{j=0}^{2n_{a}}W_{j}^{\left(m\right)}X_{j,t|t-1}^{(i)x}$$
    (37) $$P_{t|t-1}^{\left(i\right)}=\sum_{j=0}^{2n_{a}}W_{j}^{\left(c\right)}\left(X_{j,t|t-1}^{(i)x}-{\bar{X}}_{j,t|t-1}^{(i)x}\right){\left(X_{j,t|t-1}^{(i)x}-{\bar{X}}_{j,t|t-1}^{(i)x}\right)}^{T}$$
    (38) $$Y_{t|t-1}^{\left(i\right)}=h\left(X_{t-1}^{(i)x},X_{t-1}^{(i)n}\right)$$
    (39) $${\bar{y}}_{t|t-1}^{\left(i\right)}=\sum_{j=0}^{2n_{a}}W_{j}^{\left(m\right)}Y_{j,t|t-1}^{\left(i\right)}$$
    Acquire new measurement (measurement update):
    (40) $$P_{{\tilde{y}}_{t}{\tilde{y}}_{t}}=\sum_{j=0}^{2n_{a}}W_{j}^{\left(c\right)}\left(Y_{j,t|t-1}^{\left(i\right)}-{\bar{y}}_{t|t-1}^{\left(i\right)}\right){\left(Y_{j,t|t-1}^{\left(i\right)}-{\bar{y}}_{t|t-1}^{\left(i\right)}\right)}^{T}$$
    (41) $$P_{x_{t}y_{t}}=\sum_{j=0}^{2n_{a}}W_{j}^{\left(c\right)}\left(X_{j,t|t-1}^{\left(i\right)}-{\bar{x}}_{t|t-1}^{\left(i\right)}\right){\left(Y_{j,t|t-1}^{\left(i\right)}-{\bar{y}}_{t|t-1}^{\left(i\right)}\right)}^{T}$$
    (42) $$K_{t}=P_{x_{t}y_{t}}P_{{\tilde{y}}_{t}{\tilde{y}}_{t}}^{-1}$$
    (43) $${\bar{x}}_{t}^{\left(i\right)}={\bar{x}}_{t|t-1}^{\left(i\right)}+K_{t}\left(y_{t}-{\bar{y}}_{t|t-1}^{\left(i\right)}\right)$$
    (44) $${\hat{P}}_{t}^{\left(i\right)}=P_{t|t-1}^{\left(i\right)}-K_{t}P_{{\tilde{y}}_{t}{\tilde{y}}_{t}}K_{t}^{T}$$
    Sampling ${\hat{x}}_{t}^{\left(i\right)}\sim q\left(x_{t}^{\left(i\right)}|x_{0:t-1}^{\left(i\right)},y_{1:t}\right)=N\left({\bar{x}}_{t}^{\left(i\right)},{\hat{P}}_{t}^{\left(i\right)}\right)$
    Assuming ${\hat{x}}_{0:t}^{\left(i\right)}≜\left(x_{0:t-1}^{\left(i\right)},{\hat{x}}_{t}^{\left(i\right)}\right)$, ${\hat{P}}_{0:t}^{\left(i\right)}≜\left(P_{0:t-1}^{\left(i\right)},{\hat{P}}_{t}^{\left(i\right)}\right)$
    Sampling ${\hat{x}}_{t}^{\left(i\right)}\sim q\left(x_{t}^{\left(i\right)}|x_{0:t-1}^{\left(i\right)},y_{1:t}\right)=N\left({\bar{x}}_{t}^{\left(i\right)},{\hat{P}}_{t}^{\left(i\right)}\right)$
    Assuming ${\hat{x}}_{0:t}^{\left(i\right)}≜\left(x_{0:t-1}^{\left(i\right)},{\hat{x}}_{t}^{\left(i\right)}\right)$, ${\hat{P}}_{0:t}^{\left(i\right)}≜\left(P_{0:t-1}^{\left(i\right)},{\hat{P}}_{t}^{\left(i\right)}\right)$
  (ii) For *i*=1,2,…, *N*, computing the importance weight:
    (45) $$w_{t}^{\left(i\right)}\propto \frac{p\left(y_{t}|{\hat{x}}_{t}^{\left(i\right)}\right)p\left({\hat{x}}_{t}^{\left(i\right)}|x_{t-1}^{\left(i\right)}\right)}{q\left({\hat{x}}_{t}^{\left(i\right)}|x_{0:t-1}^{\left(i\right)},y_{1:t}\right)}$$
  (iii) For *i*=1,2,…, *N*, normalize the importance weight:
(2) Choosing procedure:
  We use high/low importance weight ${\tilde{w}}_{t}^{\left(i\right)}$ to increase/compress particles $\left({\hat{x}}_{0:t}^{\left(i\right)},{\hat{P}}_{0:t}^{\left(i\right)}\right)$, and get *N* random particles $\left({\tilde{x}}_{0:t}^{\left(i\right)},{\tilde{P}}_{0:t}^{\left(i\right)}\right)$.
(3) Output: As the regular PF:
  From the above procedure, we embed the UKF into the PF frame, and can easily introduce new measurements into the state estimation. Although the *a posteriori* probability density function may not follow a Gaussian distribution, we can use a Gaussian distribution to approximate every particle and keep the nonlinearity of system by UKF. Static deformation could be addressed by simply comparing the angular rates of the different IMUs. However, in practical application, static deformation should also be considered during dynamic deformation estimation. Therefore, in the proposed method, we take both static and dynamic deformation parameters into the state equation, and then use the UPF method to estimate them simultaneously. Based on the above UPF method, a flow chart of the proposed deformation estimation method is shown in Figure 6.

## 5. Simulation

**Figure 7.**
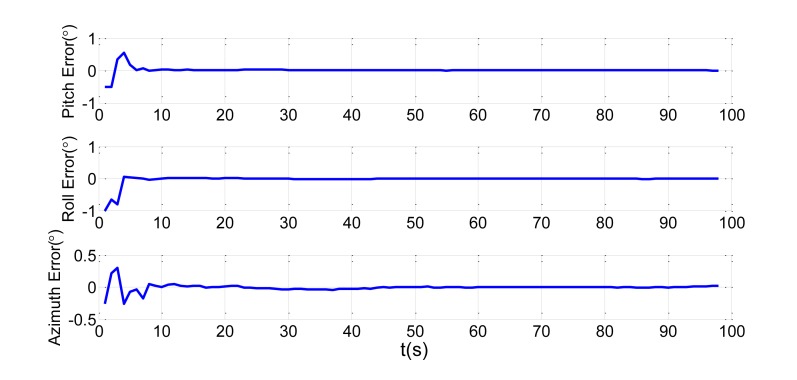
UPF estimation error of static deformation (200 particles).

**Figure 8.**
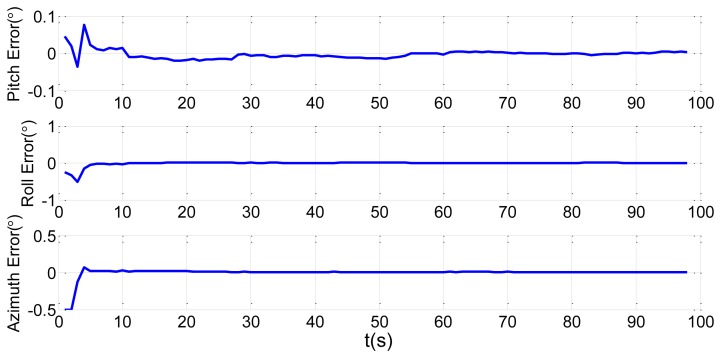
UPF estimation error of static deformation (500 particles).

In order to verify the proposed method, we choose the shipboard deformation nonlinear model from Equations (23) and (25), the large static deformation angles of pitch, roll and azimuth as 20′, 30′ and 50′, and 200 particles. The initial conditions are the same as in Section 2.2. Simulation of the UPF estimation of the deformation error is shown in Figure 7. We keep other simulation conditions the same, but choose 500 particles. The results are demonstrated in Figure 8.

It is demonstrated in Figures 7 and 8 that the UPF methods with 200 and 500 particles both converge within about 30 s. However, during simulation, the computation time of the method with 500 particles is longer than the one with 200 particles. Therefore, if the converge performance is the same, less particles should be applied to reduce the calculation burden.

**Figure 9.**
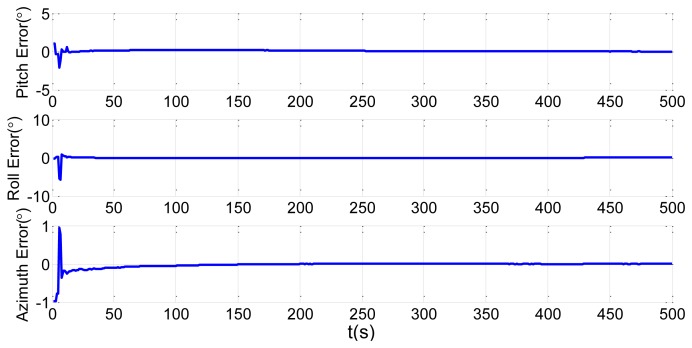
UPF estimation error of dynamic deformation (200 particles).

**Figure 10.**
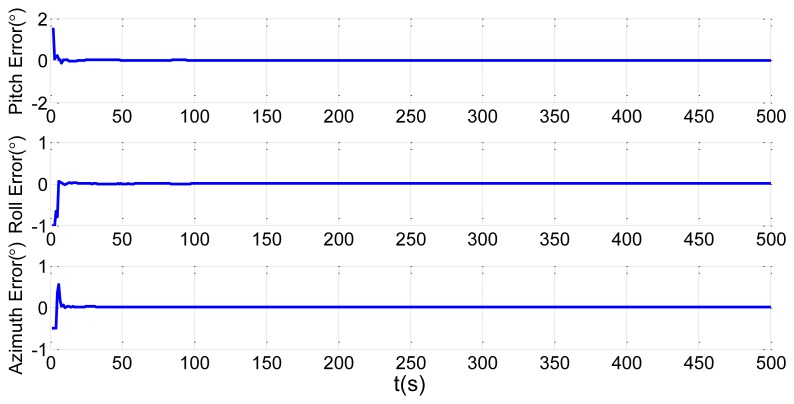
UPF estimation error of dynamic deformation (500 particles).

Under some severe conditions, large amplitude swings caused by wave and wind disturbances lead to large dynamic deformations. In order to test the effectiveness of UPF in dynamic deformation, we increase the pitch, roll, and azimuth to 5°, 4° and 2°. Choosing 200 particles, we have the estimation results of UPF in Figure 9. We then keep other simulation conditions the same but choose 500 particles. The results are shown in Figure 10.

From the results we can conclude that under the same conditions, deformation increases will cause longer convergence times. This is because of the large variation of deformation with the same particle number. Increasing the particle number will reduce the filter convergence time, but the computation burden will increase according to the theoretical analysis. For small deformations, UPF will achieve higher accuracy; for large deformations, UPF will also converge in a shorter time. The accuracy of both situations is less than 1′.

The constant drift of the gyro will be obtained by initial alignment or calibration methods at the beginning when the ship departs from the dock. Moreover, such biases are not important in shipboard deformation estimation. Therefore, even though the parameter of gyro's constant drift is in the state equation of the proposed method, we only discuss the simulation results of the shipboard deformation parameters.

**Figure 11.**
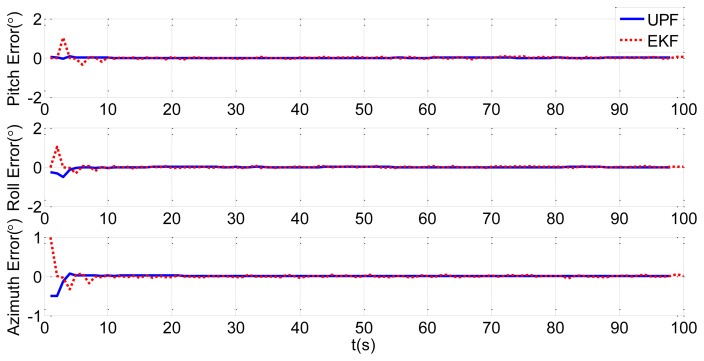
Static deformation estimation comparison of UPF and EKF.

**Figure 12.**
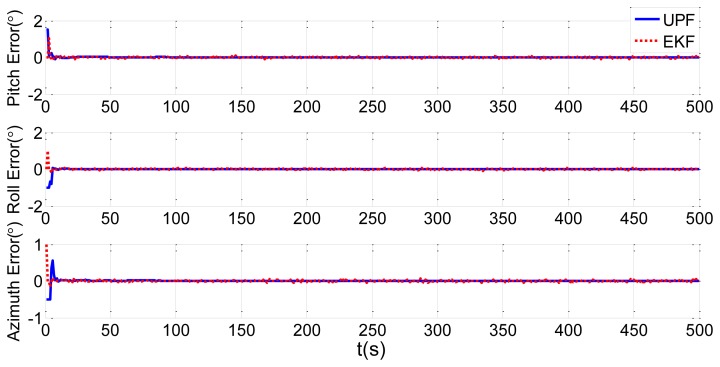
Dynamic deformation estimation comparison of UPF and EKF.

In order to verify the efficiency of the UPF method, we choose the traditional EKF method for comparison. Simulations of static and dynamic deformation are shown in Figures 11 and 12, respectively. Blue solid lines in the figures are the estimation results of the UPF method, while red dotted lines are the estimation results of the EKF method.

Figures 11 and 12 shows that the simulation results of the UPF method converge within 5 s, and the results of the EKF method converge in about 10 s, twice as long as the UPF method. From the results, we know that estimation accuracy of UPF is better than EKF, and convergence speed is faster than EKF. In the nonlinear environment, UPF makes use of the latest measurement data to produce prediction particles. When the accuracy of the measurement model is higher, the accuracy of state estimation is higher than in EKF.

**Figure 13.**
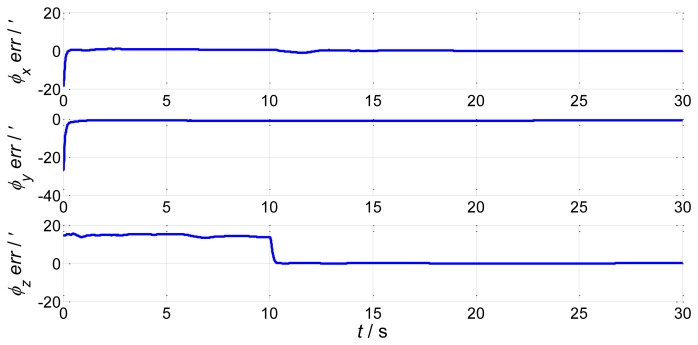
Estimation error of attitude error angles.

**Figure 14.**
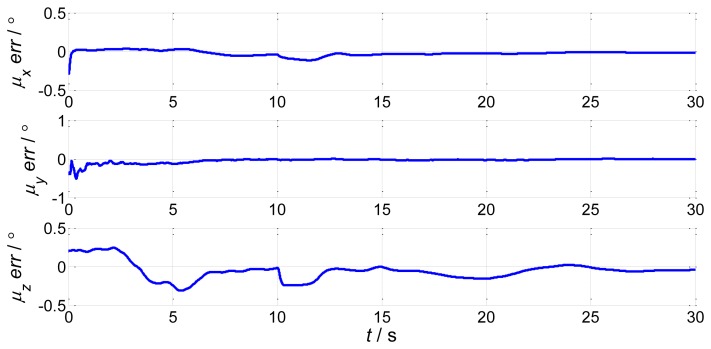
Estimation error of installation error angles.

In order to verify the efficiency of the proposed method, we present a simulation of the attitude plus angular rate match mode in aided alignment. The initial conditions are the same as Section 2.2. Assuming three compensated shipboard deformation angles are [0.5′ 9.2′ 3.4′]. Estimations of attitude error angles and installation error angles are simulated in Figures 13 and 14.

Compared to the simulations in Figures 2, 3, 4 and 5, which did not compensate the shipboard deformation, Figures 13 and 14 demonstrate that after compensation of shipboard deformation, the performance of attitude plus angular rate match mode is enhanced a lot. Estimation results converge to the true value in a short time with high accuracy.

## 6. Conclusions

An unscented particle filter method for estimation of shipboard deformation based on an inertial measurement unit is presented. In this method, we first build the nonlinear shipboard deformation model, and produce a simulation of accuracy reduction due to deformation. An attitude plus angular rate match mode is proposed to estimate the shipboard deformation during aided alignment. For the nonlinearity of the system model, a UPF method is proposed to obtain the deformation angles and compensate to aid alignment.

We use the UPF method to estimate the nonlinear model of large shipboard deformation. For different deformation angles, estimations can rapidly converge with high accuracy. The amplitude of deformation determines the convergence speed of UPF. Simulations demonstrate the estimation comparison of UPF and EKF. For static and dynamic deformation angles, UPF, which is a more effective estimation method of deformation, has higher estimation accuracy and converges more rapidly than EKF. After shipboard deformation compensation, simulation shows the accuracy of aided alignment is also increased. In future work, multiple IMUs should be considered for the ship deformation measurement. Multiple spots will provide full and effective ship deformation measurements.
